# The alterations in multiple neurophysiological procedures are associated with frailty phenotype in older adults

**DOI:** 10.3389/fnagi.2023.1063322

**Published:** 2023-03-28

**Authors:** Xin Jiang, Junhong Zhou, Chengyuan Yu, Wenbo Chen, Baofeng Huang, Yurong Chen, Lilian Zhong, Yi Guo, Qingshan Geng, Yurun Cai

**Affiliations:** ^1^Department of Geriatrics, Shenzhen People’s Hospital, Shenzhen, Guangdong, China; ^2^The Second Clinical Medical College, Jinan University, Shenzhen, Guangdong, China; ^3^The First Affiliated Hospital, Southern University of Science and Technology, Shenzhen, Guangdong, China; ^4^Hinda and Arthur Marcus Institute for Aging Research, Hebrew SeniorLife, Roslindale, MA, United States; ^5^Division of Gerontology, Beth Israel Deaconess Medical Center, Boston, MA, United States; ^6^Harvard Medical School, Boston, MA, United States; ^7^Department of Neurology, Shenzhen People’s Hospital, Shenzhen, Guangdong, China; ^8^Shenzhen Bay Laboratory, Shenzhen, Guangdong, China; ^9^Department of Health and Community Systems, University of Pittsburgh School of Nursing, Pittsburgh, PA, United States

**Keywords:** neurophysiological alterations, multisystem comorbidities, vascular function, frailty phenotype, frail status

## Abstract

**Background:**

Older adults oftentimes suffer from the conditions in multiple physiologic systems, interfering with their daily function and thus contributing to physical frailty. The contributions of such multisystem conditions to physical frailty have not been well characterized.

**Methods:**

In this study, 442 (mean age = 71.4 ± 8.1 years, 235 women) participants completed the assessment of frailty syndromes, including unintentional weight loss, exhaustion, slowness, low activity, and weakness, and were categorized into frail (≥3 conditions), pre-frail (1 or 2 conditions), and robust (no condition) status. Multisystem conditions including cardiovascular diseases, vascular function, hypertension, diabetes, sleep disorders, sarcopenia, cognitive impairment, and chronic pain were assessed. Structural equation modeling examined the interrelationships between these conditions and their associations with frailty syndromes.

**Results:**

Fifty (11.3%) participants were frail, 212 (48.0%) were pre-frail, and 180 (40.7%) were robust. We observed that worse vascular function was directly associated with higher risk of slowness [standardized coefficient (SC) = −0.419, *p* < 0.001], weakness (SC = −0.367, *p* < 0.001), and exhaustion (SC = −0.347, *p* < 0.001). Sarcopenia was associated with both slowness (SC = 0.132, *p* = 0.011) and weakness (SC = 0.217, *p* = 0.001). Chronic pain, poor sleep quality, and cognitive impairment were associated with exhaustion (SC = 0.263, *p* < 0.001; SC = 0.143, *p* = 0.016; SC = 0.178, *p* = 0.004, respectively). The multinomial logistic regression showed that greater number of these conditions were associated with increased probability of being frail (odds ratio>1.23, *p* < 0.032).

**Conclusion:**

These findings in this pilot study provide novel insights into how multisystem conditions are associated with each other and with frailty in older adults. Future longitudinal studies are warranted to explore how the changes in these health conditions alter frailty status.

## Introduction

Frailty phenotypes are one of the conditions that are commonly observed in older adult population and have been linked to multiple health-related events/issues in this population (Fried et al., 2001; Collard et al., 2012). Older frail adults, for example, are with greater likelihood of falls, and suffer from loss of functional independence (e.g., disabled mobility) as compared to those who are not frail (Cheng and Chang, 2017). The appropriate characterization of frailty status and understanding of the relationships between its underlying neurophysiological components are thus of great importance for clinicians, researchers, and senior care givers, which can ultimately help optimize the management and rehabilitation programs aiming to minimize the burden of being frail in older adult population.

Frailty is oftentimes defined and characterized by assessing multiple “phenotypes” (Fried et al., 2001; Bandeen-Roche et al., 2006; Rockwood and Mitnitski, 2007). For example, Fried’s frailty phenotype, one of most widely used criteria in clinical practice (Fried et al., 2001), characterizes five typical phenotypes, that is, unintentional weight loss, low energy or self-reported exhaustion, low physical activity, slowness, and weakness. The severity of frailty is then categorized by simply summing the number of these phenotypes (e.g., “frail” is defined as the presence of at least three phenotypes). Although these protocols to characterize frailty phenotypes have been validated and implemented in clinics, little is known about the associations between the underlying neurophysiological procedures and these frailty phenotypes (Savva et al., 2013; Donoghue et al., 2020).

Recent studies have emerged to show that the frailty phenotypes may arise from decreased homeostatic reserve/resilience across multiple bio-neurophysiological procedures in aging (Fried et al., 2021), and thus these seemingly distinct phenotypes may share the same complex underlying pathophysiological components, such as cognitive decline, cardiovascular abnormalities, and sarcopenia (Buchman et al., 2007; Kelaiditi et al., 2013; Cesari et al., 2014; Robertson et al., 2014; Vetrano et al., 2018). Therefore, the explicit characterization of the complex interactions between those neurophysiological procedures, and their relationships to those frailty phenotypes in older adults can provide novel insights into the pathophysiology of frailty.

In this study consisting of a group of community-dwelling older adults, the frailty phenotypes were assessed following Fried frailty phenotype. Multiple neurophysiological conditions (e.g., cardiovascular diseases, sarcopenia, diabetes, and chronic pain) and functions (e.g., cognitive function, sleep quality, and vascular function) that are potentially related to frailty syndromes were carefully assessed. We then examined the inter-relationships between these characteristics and their relationships to each frailty criterion and the overall frailty phenotype.

## Materials and methods

### Participants

The participants were recruited *via* the search of an electronic clinical data repository in the Department of Geriatrics, Shenzhen People’s Hospital. This repository was initiated on 1 January 2020, consisting of older adults who had clinical visit for annual physical exam or primary care, without any emergent or severe clinical conditions. The cut-off date of the search was set on 1 January 2022. Therefore, older adults who had a clinical visit between 1 January 2020, and 1 January 2022, and expressed the interests in participating in future studies were contacted. The inclusion criteria were older adults with (1) age ≥60 years at the visit of this study, and (2) the ability to walk for at least 30 s without personal assistance. The exclusion criteria were: (1) diagnosis of dementia or other overt neurological diseases (e.g., Parkinson’s disease), (2) diagnosis of terminal disease (e.g., cancer), (3) history of brain trauma or injury, (4) hospitalization within the past 6 months, (5) uncontrolled hypertension, (6) chronic kidney disease, dyslipidemia, (7) overt psychological or mood conditions (e.g., depression and anxiety), (8) undergoing or received cardiac surgery, and (9) inability to understand the study protocol. All experimental protocols were approved by the local Institutional Review Board and carried out in accordance with the guidelines in the Declaration of Helsinki. All the participants provided written consent in order to participate in this study.

### Study protocol

After screening, a total of 442 eligible participant completed two study visits separated by 1 day. On the first visit, they completed a series of questionnaires to assess the demographics [e.g., age, sex, and body mass index (BMI)], health behaviors (e.g., smoking and alcohol), and the assessments of frailty, sleep quality, cognitive function, sarcopenia, and chronic pain. On the second visit, they completed assessments related to cardiovascular function, including continuous beat-to-beat BP assessment, and the assessment of arterial stiffness, in the same laboratory under the administration of at least one research staff member to ensure the safety of participants.

### Study visit 1

#### Assessment of frailty

Frailty was assessed by Fried Frailty Phenotype Criteria (Fried et al., 2001). Five syndromes were assessed, including unintentional weight loss, low energy or self-reported exhaustion, low physical activity, slowness as assessed by slowed walking speed, and weakness as measured by low grip strength. One research staff member administrated this assessment by providing the instructions and description of the assessment. Based upon the results of assessments and self-reports, the severity of frailty was categorized into three stages: robust (i.e., no criterion was presented), pre-frail (i.e., one or two criteria were presented), and frail (i.e., three or more criteria were presented).

#### Sleep quality

The sleep quality was measured using Chinese version of Pittsburgh Sleep Quality Index (PSQI) (Tsai et al., 2005). The total score of PSQI ranged from 0 to 21, and a score greater than 5 was defined as having poor sleep quality.

#### Cognitive function

The Chinese version of mini-mental state examination (MMSE) was used to assess general cognitive function of each participant (Katzman et al., 1988). The total score of MMSE ≤24 was defined as having cognitive impairment and the status of it (i.e., yes or no) was used in the following analysis.

#### Sarcopenia

Sarcopenia was identified by measuring the skeletal muscle mass (ASM) *via* bioelectrical impedance analysis (BIA) (Gonzalez et al., 2018). When a man had the value of ASM ≤7.0 kg/m^2^ or a woman had ASM ≤5.7 kg/m^2^ as measured by BIA, he/she was identified to have sarcopenia. The status of sarcopenia (i.e., yes or no) was used in the following analyses.

#### Chronic pain

The chronic pain is defined as participant experienced pain lasting for at least six months (Russo and Brose, 1998). The severity of pain was then measured using Numeric Pain Rating Scale (NPRS) (Farrar et al., 2001; Kahl and Cleland, 2005). Greater NPRS reflected worse pain.

#### Hypertension

Hypertension was carefully assessed on the previous clinical visit for each participant by an experienced clinician. The information was then extracted from the clinical records and included in this study. Specifically, hypertension was defined as systolic blood pressure (SBP) ≥140 mmHg and/or diastolic blood pressure (DBP) ≥90 mmHg by measuring the brachial artery of right arm using a sphygmomanometer.

#### Diabetes

Diabetes was diagnosed on the previous clinical visit following a comprehensive diagnostic protocol, in which participants who had self-reported signs of diabetes and those with glycated hemoglobin (HbA1c) level ≥6.5% was identified as diabetes.

#### Cardiovascular disease

Participant’s cardiovascular disease (CVD) history (e.g., coronary heart disease and chronic heart failure) was obtained from their medical records.

### Study visit 2

#### Vascular function assessment

We measured the complexity of beat-to-beat BP fluctuation (i.e., BP complexity), and the arterial stiffness using brachial-ankle pulse wave velocity (baPWV) (Yamashina et al., 2002). In previous studies from our group and others, both BP complexity and baPWV were closely associated with the frailty status (Amarasekera et al., 2021; Jiang et al., 2022a).

#### BP complexity

Blood pressure is determined by multiple elements, such as the systemic vascular resistance (Stefadouros et al., 1973), and regulated by neural and hormonal feedback procedures, such as baroreceptors, over multiple, not single, and temporospatial scales (Dauphinot et al., 2013). Therefore, the dynamics of the beat-to-beat BP fluctuation is complex, and the complexity in BP fluctuation contains important information of the neurophysiology in vascular system. Studies have shown that the BP complexity, which can be quantified by using multiscale entropy (MSE) (Costa et al., 2002, 2005), is an appropriate marker to capture the subtle functional changes vascular system and closely associated with frailty (Jiang et al., 2021, 2022a,b). We here followed the protocol that was used in the previous work to record the continuous beat-to-beat BP series of 10–15 min and quantified the complexity of systolic (SBP) and diastolic (DBP) BP using MSE.

To measure the beat-to-beat BP series, after rest following the HbA1c test, each participant completed the BP assessment while sitting in a quiet assessment room with one study staff member. During the assessment, the participant was instructed to not talk and to keep motionless (e.g., not to move the arms). The objects which may interfere with testing, such as mobile phone, were stored in a box outside the room. The continuous beat-to-beat SBP and DBP series were recorded using Finometer PRO system (Finapres Medical Systems B.V., Netherlands) at the middle finger of left hand for 10–15 min while the participant was in the supine position. The sampling frequency was 100 Hz. All the BP recordings consisted of at least 700 continuous beats. The BeatScope software package (Finapres Medical Systems B.V., Netherlands) was then used to calculate the BP values of each beat.

For the pre-processing of BP series, we followed the procedure that has been used in our previous studies (Jiang et al., 2021, 2022b). Specifically, the outliers in BP series were determined as those points of the value greater or lower than mean ± two times of standard deviations (SD) of the series. These outliers were then interpolated by the mean of this series. The pre-processed BP series of 700 sampling points were then used to calculate BP complexity.

The complexity of SBP and DBP series was quantified using MSE, a well-developed technique that quantifies the entropy or recurrence in physiologic series over different temporal or spatial scales. Specifically, the preprocessed BP series was first “coarse-grained” from Scale 1 to 5. In this coarse graining procedure, the original series was divided into non-overlapping windows of length of 1 (i.e., Scale 1) to 5 (i.e., Scale 5) sampling points. The coarse-grained series at Scale 1 was thus the raw series consisting of 700 points, and that at Scale 5 was constructed by averaging every five non-overlapping points [e.g., the first point of the coarse-grained series at Scale 5 was obtained by averaging from Point 1 to 5 in the original series and the second was obtained by averaging from Point 6 to 10 of the original series Costa et al., 2002)], consisting of 140 points (i.e., 700 points/5). We then calculated the sample entropies of each “coarse-grained” series by following the established procedure, that is, the negative natural logarithm of the conditional probability that a series, having repeated itself within a tolerance r for m points, will also repeat itself for m + 1 points without self-matches (Costa et al., 2005). The tolerance r of 0.15 and number of matching points (m) of 2 were used. The number of points in the coarse-grained BP series at largest scale (i.e., Scale 5) was 140, which was greater than that is recommended for reliable estimation of sample entropy in this parameter setting we used (Costa et al., 2005). Specifically, to obtain an appropriate estimate of entropy, the number of data points in the largest-scale coarse-grained series should at least be 10*^m^* to 17*^m^*; therefore, the appropriate number on the largest scale would be 100∼289 when using m of 2. We thus chose to calculate the MSE over Scale 1 to 5 following our previous work (Jiang et al., 2021, 2022a,b) and the well-established procedure (Costa et al., 2005). The complexity of SBP and DBP was then defined as the averaged entropy across five scales, and lower averaged entropy reflected lower complexity.

#### Assessment of arterial stiffness

Following the testing procedures as suggested (Yamashina et al., 2002; Turin et al., 2010; Thijssen et al., 2011), the arterial stiffness was assessed by measuring the left- and right-side brachial-ankle pulse wave velocity (baPWV) (Omron, Kyoto, Japan) when participants were in resting state. One research staff administrated this assessment by providing the instructions of the well-established testing procedure to guide the participants and ensure the quality of the measurement. The averaged baPWV was used in the following analysis.

### Statistical analysis

Means and SDs were used to describe continuous variables and frequency and percentage were used to describe categorical variables. Sociodemographic and multisystem conditions were summarized for the entire cohort and within each frailty group: robust, pre-frail, and frail. To examine the difference in these characteristics between groups, we used Chi-square tests for categorical variables and analysis of variance (ANOVA) tests for continuous variables. The factor of each model was frailty status.

To examine the inter-relationships between those measured neurophysiological characteristics, and their association/contribution to frailty phenotypes, structural equation modeling (SEM) analysis was used. Latent factor of vascular function was constructed using confirmatory factor analysis by baPWV, SBP, and DBP complexity. The variance of the latent factor was constrained to be 1. In addition to the relative associations to frail syndromes, we also examined the associations between these outcomes, including vascular function, chronic pain, sarcopenia, diabetes, sleep disorder, and cognitive impairment, using Bayesian estimation. The results of both unadjusted model and model adjusted for age, sex, and BMI were obtained.

To examine the relationship of the neurophysiological characteristics to the odds of pre-frail and frail status, multinomial logistic regression models were used to measure the odds ratios (ORs) of pre-frail and frail status by using the robust group as reference. The effects of different conditions (e.g., cognitive decline and diminished sleep quality), BP complexity, PWV, and the number of conditions on the ORs were examined in separate models. All models were adjusted for age, sex, and body mass index, and maximum likelihood estimation was used. We then conducted the receiver operating characteristic (ROC) curve analysis (Hand and Till, 2001). Multi-level area under the curve (AUC) of each outcome were derived from these multinomial models.

The significance level α was set as 0.05. Descriptive statistical analyses were conducted using SAS 9.4 (SAS Institute, Cary, NC, USA). The SEM analysis was performed using Mplus 8.7 (Muthén & Muthén, Los Angeles, CA, USA).

## Results

**FIGURE 1:**
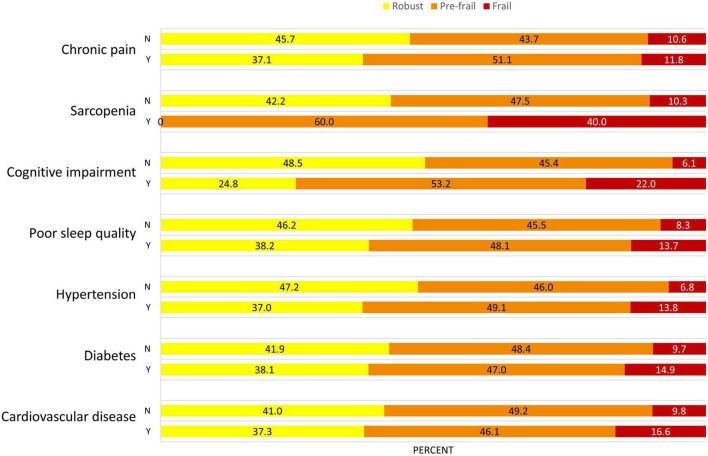
Prevalence of frailty status in different health conditions in this population. The proportion of frailty (i.e., frail in red, pre-frail in orange) is greater in participants who suffering from these health conditions. Y, yes; N, no.

**FIGURE 2:**
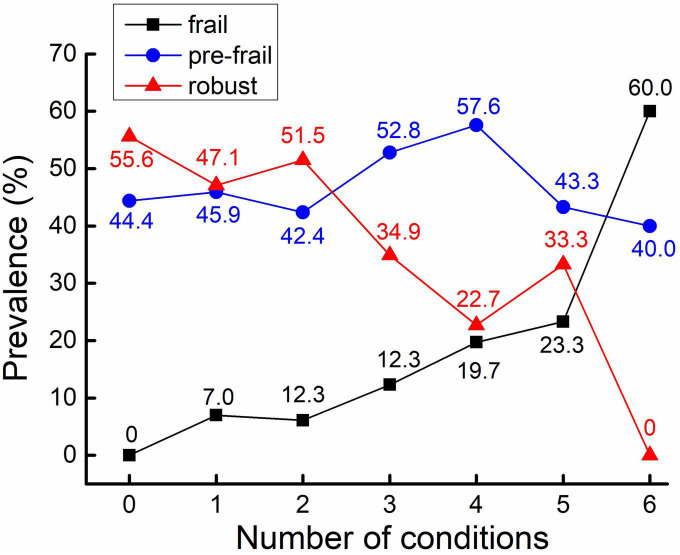
Prevalence of frailty status in different numbers of health conditions. The prevalence (as assessed by percentage) of frail participants is increased along with the increase of the number of health conditions (in black).

Among a total of 442 participants, 50 (11.3%) participants were frail, 212 (48.0%) were pre-frail, and 180 (40.7%) were robust. The mean age was 71.1 (SD = 8.0) years, and 235 participants (53.2%) were women (Table 1). The average education years were 9.9 ± 4.7 years, and the BMI was 24.4 ± 3.8 in all participants. For frailty phenotypes, 29 (7.4%) participants had unintentional weight loss, 47 (11.4%) had slowness, 211 (48.2%) had weakness, 94 (21.3%) had exhaustion, and 39 (8.9%) had low activity. Meanwhile, 102 (24.5%) had cardiovascular disease, 134 (30.3%) had diabetes, 281 (63.6%) had hypertension, 233 (59.9%) had poor sleep quality, 109 (29.4%) had cognitive impairment, 15 (3.4%) had sarcopenia, and 237 (54.4%) reported chronic pain. The average number of these conditions was 2.4 (SD = 1.2) for the entire cohort. Specifically, 18 (4.1%) participants were without any of these conditions, 85 (19.2%) with one condition, 132 (29.9%) with two conditions, 106 (24.0%) with three conditions, 66 (14.9%) with four conditions, 30 (6.8%) with five conditions, and five (1.1%) with six conditions. ANOVA models showed significant differences in multiple characteristics between groups. Specifically, compared to robust group, significantly elevated decrease in SBP and DBP complexity, and increase in PVW and number of multisystem conditions was observed from pre-frail to frail group (*p* < 0.001). Additionally, compared to robust group, elevated increase in age, BMI, the prevalence of hypertension, cognitive impairment, and sarcopenia were also associated with frailty status (*p* < 0.01; Table 1). No such significant difference in the prevalence of CVD, diabetes, poor sleep quality and chronic pain was observed between groups (*p* = 0.052∼0.652). The prevalence of frailty status by these seven health conditions was shown in Figure 1. Overall, a higher prevalence of being frail and a lower prevalence of being robust were associated with increased number of these conditions, with a dramatic increase from 5 to 6 conditions (Figure 2).

**TABLE 1:** Sociodemographic and health characteristics in entire cohort and within each group of frailty severity.

Characteristics *n* (%) or mean ± SD	Total (*n* = 442)	Robust (*n* = 180)	Pre-frail (*n* = 212)	Frail (*n* = 50)	*p*-Value^a^
Age (years)	71.1 ± 8.0	68.2 ± 6.4	71.4 ± 7.6	80.5 ± 7.7	**<0.001**
Sex					0.652
Male	207 (46.8)	91 (50.6)	89 (42.0)	27 (54.0)	
Female	235 (53.2)	89 (49.4)	123 (58.0)	23 (46.0)	
Education (years)	9.9 ± 4.7	10.5 ± 4.5	9.3 ± 4.8	9.3 ± 5.1	**0.02**
Body mass index	24.4 ± 3.8	24.3 ± 3.6	24.8 ± 3.9	22.6 ± 3.4	**0.003**
SBP complexity	1.43 ± 0.27	1.50 ± 0.26	1.40 ± 0.26	1.25 ± 0.22	**<0.001**
DBP complexity	1.30 ± 0.29	1.40 ± 0.27	1.27 ± 0.29	1.11 ± 0.28	**<0.001**
PWV (m/s)	17.83 ± 4.12	16.6 ± 2.9	18.15 ± 4.29	21.07 ± 5.29	**<0.001**
Number of health conditions	2.51 ± 1.31	2.21 ± 1.20	2.58 ± 1.30	3.32 ± 1.39	**<0.001**
Cardiovascular disease	102 (24.5)	38 (22.8)	47 (23.3)	17 (35.4)	0.162
Diabetes	134 (30.3)	51 (28.3)	63 (29.7)	20 (40.0)	0.187
Hypertension	281 (63.6)	104 (57.8)	138 (65.1)	39 (78.0)	**0.008**
Poor sleep quality (PSQI >7)	233 (59.9)	89 (55.3)	112 (61.2)	32 (71.1)	0.052
Cognitive impairment (MMSE ≤24)	109 (29.4)	27 (17.5)	58 (32.8)	24 (60.0)	**<0.001**
Sarcopenia^b^	15 (3.4)	0	9 (4.3)	6 (12.0)	**<0.001**
Chronic pain	237 (54.4)	88 (49.2)	121 (58.2)	28 (57.1)	0.120

SD, standard deviation; SBP, systolic blood pressure; DBP, diastolic blood pressure; PWV, pulse wave velocity.

^a^Mantel–Haenzel Chi-square test for trend (1 d.f.) for categorical variables and ANOVA test for continuous variables. Bolded *p*-values indicate statistically significant results.

### The inter-relationships between the underlying neurophysiological characteristics and their associations to different frailty phenotypes

**FIGURE 3:**
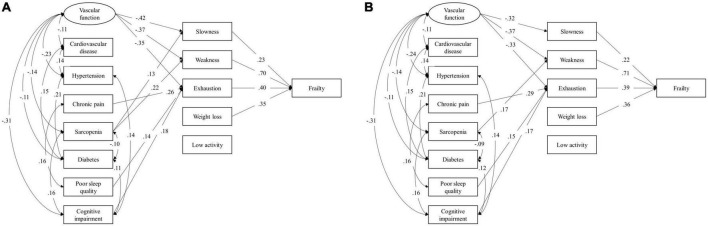
Structural equation model (SEM) unadjusted **(A)** and adjusted **(B)** for age, sex, and body mass index for the associations between health conditions and frailty phenotypes. Latent factor of vascular function was constructed using confirmatory factor analysis by baPWV, SBP, and DBP complexity. The variance of the latent factor was constrained to be 1. Bayesian estimation was used to estimate path coefficients. All loadings are standardized coefficients and significant at *p* < 0.05. Non-significant paths are omitted in this diagram. BaPWV, brachial-ankle pulse wave velocity; SBP, systolic blood pressure; DBP, diastolic blood pressure.

Latent factor of vascular function in SEMs was constructed by baPWV, SBP, and DBP complexity, with factor loadings as 0.39, 0.80, and 0.84, respectively. The unadjusted model showed that worse vascular function was directly associated with higher risk of slowness [standardized coefficient (SC) = −0.419, 95% confidence interval (CI) = −0.598, −0.220, *p* < 0.001], weakness (SC = −0.367, 95% CI = −0.504, −0.220, *p* < 0.001), and exhaustion (SC = −0.347, 95% CI = −0.495, −0.182, *p* < 0.001; Figure 3A). Chronic pain was associated with exhaustion (SC = 0.263, 95% CI = 0.139, 0.380, *p* < 0.001). Sarcopenia was associated with both slowness (SC = 0.132, 95% CI = 0.021, 0.240, *p* = 0.011), and weakness (SC = 0.217, 95% CI = 0.077, 0.375, *p* = 0.001). Both poor sleep quality and cognitive impairment were associated with exhaustion (SC = 0.143, 95% CI = 0.013, 0.270, *p* = 0.016; SC = 0.178, 95% CI = 0.048, 0.301, *p* = 0.004, respectively). No direct association of cardiovascular disease, hypertension, and diabetes to any frailty phenotypes was observed, but they were significantly correlated with vascular function and other conditions. Additionally, among five frail phenotypes, slowness (SC = 0.234, 95% CI = 0.096, 0.371), weakness (SC = 0.696, 95% CI = 0.605, 0.783), exhaustion (SC = 0.403, 95% CI = 0.287, 0.524), and weight loss (SC = 0.348, 95% CI = 0.251, 0.447) were significantly associated with frailty status (Figure 3A). After adjusting for age, sex, and BMI, similar results to unadjusted model were observed, except for the path from sarcopenia to slowness, which lost significance in the adjusted model (Figure 3B).

### The relationship between the neurophysiological characteristics and the odds of pre-frail and frail status

Multinomial logistic regression models showed that participants with one unit of lower SBP or DBP complexity was associated with 72% (OR = 0.28, 95% CI = 0.10, 0.83, *p* = 0.021) or 77% (OR = 0.23, 95% CI = 0.08, 0.60, *p* = 0.003) greater odds of being pre-frail, and 91% (OR = 0.09, 95% CI = 0.01, 0.57, *p* = 0.011) or 90% (OR = 0.10, 95% CI = 0.02, 0.50, *p* = 0.006) greater odds of being frail, respectively (Table 2). Participants with 10 m/s higher PWV had more than doubled odds of being pre-frail (OR = 2.63, 95% CI = 1.18, 5.85, *p* = 0.018) and over tripled odds of being frail (OR = 3.26, 95% CI = 1.11, 9.59, *p* = 0.032). Participants with poor sleep quality was associated with more than doubled odds of being frail (OR = 2.66, 95% CI = 1.07, 6.60, *p* = 0.035). Those with cardiovascular disease (OR = 2.45, 95% CI = 1, 6.01, *p* = 0.050) or hypertension (OR = 2.43, 95% CI = 0.93, 6.32, *p* = 0.070) had more than two times of odds of being frail, but such associations were only marginally significant. Older adults with cognitive impairment had 80% higher odds of being pre-frail, which was marginally significant (OR = 1.80, 95% CI = 0.98, 3.32, *p* = 0.059), and three times the odds of being frail (OR = 2.98, OR = 1.13, 7.85, *p* = 0.027). Additionally, it was observed that one more condition had elevated 23% (OR = 1.23, 95% CI = 1.02, 1.48, *p* = 0.032) and 83% (OR = 1.83, 95% CI = 1.33, 2.52, *p* < 0.001) higher odds of being pre-frail and frail, respectively. The results of ROC curve analysis showed comparable AUCs among all the multinomial models, with a range from 0.747 (i.e., PWV) to 0.770 for number of health conditions (Table 2).

**TABLE 2:** Multinomial logistic regression for the association between health conditions and frailty status after adjusted for age, sex, and body mass index.

IV	DV	OR	95% CI	*p*-Value	Multilevel AUC
SBP complexity	Robust	Ref.	Ref.	Ref.	0.755
	Pre-frail	**0.28**	**0.10–0.83**	**0.021**	
	Frail	**0.09**	**0.01–0.57**	**0.011**	
DBP complexity	Robust	Ref.	Ref.	Ref.	0.756
	Pre-frail	**0.23**	**0.08–0.60**	**0.003**	
	Frail	**0.10**	**0.02–0.50**	**0.006**	
PWV (per 10 m/s)	Robust	Ref.	Ref.	Ref.	0.747
	Pre-frail	**2.63**	**1.18–5.85**	**0.018**	
	Frail	**3.26**	**1.11–9.59**	**0.032**	
Number of health conditions	Robust	Ref.	Ref.	Ref.	0.770
	Pre-frail	**1.23**	**1.02–1.48**	**0.032**	
	Frail	**1.83**	**1.33–2.52**	**<0.001**	
Cardiovascular disease	Robust	Ref.	Ref.	Ref.	0.765
	Pre-frail	1.05	0.61–1.80	0.873	
	Frail	2.45	1.00–6.01	0.050	
Diabetes	Robust	Ref.	Ref.	Ref.	0.752
	Pre-frail	1.03	0.62–1.71	0.901	
	Frail	1.64	0.69–3.91	0.263	
Hypertension	Robust	Ref.	Ref.	Ref.	0.756
	Pre-frail	1.07	0.66–1.73	0.798	
	Frail	2.43	0.93–6.32	0.070	
Poor sleep quality (PSQI >7)	Robust	Ref.	Ref.	Ref.	0.758
	Pre-frail	1.43	0.86–2.37	0.164	
	Frail	**2.66**	**1.07–6.60**	**0.035**	
Cognitive impairment (MMSE ≤24)	Robust	Ref.	Ref.	Ref.	0.761
	Pre-frail	1.80	0.98–3.32	0.059	
	Frail	**2.98**	**1.13–7.85**	**0.027**	
Sarcopenia^a^	Robust	Ref.	Ref.	Ref.	0.758
	Pre-frail	–	–	–	
	Frail	–	–	–	
Chronic pain	Robust	Ref.	Ref.	Ref.	0.754
	Pre-frail	1.54	0.96–2.47	0.075	
	Frail	2.00	0.88–4.57	0.101	

IV, independent variable; DV, dependent variable; OR, odds ratio; CI, confidence interval; SBP, systolic blood pressure; DBP, diastolic blood pressure; PWV, pulse wave velocity.

^a^There is a quasi-compete separation in data points. The maximum likelihood estimate may not exist. Significant ORs and their 95% CI have been highlighted in bold.

## Discussion

We here examined the inter-relationships across health conditions and functions in multiple neurophysiological systems (e.g., vascular system) and their contributions to each of frail phenotypes in a group of well-characterized older adults. Older adults with poorer vascular function, cognitive function, and/or sleep quality, as well as increased number of multisystem conditions had greater odds of being pre-frail and/or frail. More importantly, these characteristics were inter-correlated with each other and directly or indirectly contribute to different frailty phenotypes, indicating that those seemingly distinct frail phenotypes may share the same underlying neurophysiological function (e.g., slowness, weakness, exhaustion, and weight loss were all directly related to vascular function). These observations are worthwhile to be considered in future clinical and rehabilitative practice for older frail population.

We here provided novel evidence to the inter-relationships between those multisystem conditions and their relative contributions to frailty phenotypes. Vascular function, for example, is directly associated with four frailty phenotypes, and with the abnormalities in metabolic and musculoskeletal systems. On the other hand, CVD is one important factor to the development of frailty (Lipsitz, 2004; Stewart, 2019), but our results showed that CVD is not a significant *direct* factor to any of frail phenotypes when other factors included, indicating that CVD may have important contribution to the development of frailty *via* its interaction with other conditions. Taken together, our observations may provide preliminary knowledge on the important underlying factors and their interactions that cannot be ignored in frailty. This may ultimately help optimize the efficacy of clinical tools (such as including those direct factors but not those indirect factors) for the screening of frailty phenotypes.

Meanwhile, we observed that compared to the non-frail group, those with greater number of multisystem conditions had significantly increased odds of being pre-frail or frail, and different phenotypes may share the same conditions, and the AUC of ROC analyses showed acceptable (i.e., >0.7) performance of using these conditions to identify individuals in pre-frail and frail from those in robust group. These are consistent with findings in previous studies (Atkinson et al., 2007; Afilalo et al., 2009; Martin et al., 2013; Montero-Odasso et al., 2017). For example, the activation of the supraspinal prefrontal network is critical to the regulation of gait, in addition to peripheral musculoskeletal condition (e.g., sarcopenia) (Mirelman et al., 2017; Zhou et al., 2021). Meanwhile, Fried et al. (2021) proposed a theory that frailty may be a consequence that emerges when aging or age-related conditions alter the regulation of multiple interconnected biophysiological systems, disrupting the complex dynamics of those systems. Following this idea, by uniquely quantifying the complex dynamics of vascular system using BP complexity, we here observed that lower BP complexity is significantly associated with greater odds of being pre-frail and frail. This is also consistent with our recent study showing that compared to robust, older adults in pre-frail and frail had significantly lower BP complexity, and more interestingly, such BP complexity mediated the relationship between arterial stiffness and frailty (Jiang et al., 2022a). Additionally, numerous studies showed that this complexity metric, which characterizing the dynamics of a given neurophysiological procedure [e.g., the heartbeat fluctuation (Toosizadeh et al., 2021)] over multiple scales of time or space, can better capture subtle changes in this system that cannot be appropriately captured by traditional single-scale measures (e.g., mean or variability) (Lipsitz, 2002; Thijssen et al., 2011; Manor and Lipsitz, 2013; Jiang et al., 2021, 2022b). This complexity metric can thus provide insights into the pathology of health conditions [e.g., frailty, the absence of autonomic surveillance of the cardiovascular system (Moen et al., 2022)]. Taken together, the results suggest that in addition to the functionality of one given procedure itself, the multiscale interactions across neurophysiological procedures are one critical factor contributing to the pathology of frailty, which should be carefully considered in future studies.

Given the limitation of cross-sectional analysis in this pilot study with relatively small sample size of participants, further studies with large sample size are needed to examine and confirm the observations in this study. Though the processing protocol of BP series have been used in a series of our previous studies, it is worthwhile to perform a specific validation study to establish a standardized protocol for the estimation of the BP complexity. The longitudinal analysis is also highly demanded to explore the causal relationships between those multisystem conditions and the development of these frail phenotypes as we assessed in this work. Meanwhile, Marinus et al. (2021) showed that the prevalence of frailty may only be associated with some specific CVDs (e.g., heart failure); therefore, future studies may explore the inter-relationships of specific types of CVD to other conditions and frail phenotypes. Previous studies showed the mood issues, such as depression (Soysal et al., 2017), were associated with frailty, which was not assessed in this “mentally-healthy” cohort. It should also be noted that no significant relationship of these conditions to weight loss and low activity was observed here, potentially due to the small number of participants who had these two phenotypes. Future studies consisting of similar numbers between five frail phenotypes are needed to examine and confirm the observations in this work. Nevertheless, this study provides novel insights into the associations of multiple neurophysiological conditions and their inter-relationships to clinically assessed frail phenotypes and status in older adults.

## Data availability statement

The raw data supporting the conclusions of this article will be made available by the authors, without undue reservation.

## Ethics statement

The studies involving human participants were reviewed and approved by the Shenzhen People’s Hospital. The patients/participants provided their written informed consent to participate in this study.

## Author contributions

XJ, JZ, and YCa designed the study. XJ, CY, WC, BH, YCh, and LZ collected the data. XJ, JZ, YG, QG, and YCa analyzed the data and performed the statistical analyses. XJ, JZ, YG, QG, and YCa interpreted the results and drafted the manuscript. All authors contributed to the article and approved the final version.
